# Graduated compression stockings as adjuvant to pharmaco-thromboprophylaxis in elective surgical patients (GAPS study): randomised controlled trial

**DOI:** 10.1136/bmj.m1309

**Published:** 2020-05-13

**Authors:** Joseph Shalhoub, Rebecca Lawton, Jemma Hudson, Christopher Baker, Andrew Bradbury, Karen Dhillon, Tamara Everington, Manjit S Gohel, Zaed Hamady, Beverley J Hunt, Gerrard Stansby, David Warwick, John Norrie, Alun H Davies

**Affiliations:** 1 Department of Surgery and Cancer, Imperial College London & Imperial Vascular Unit, Imperial College Healthcare NHS Trust, London W6 8RF, UK; 2 Health Services Research Unit, University of Aberdeen, Health Sciences Building, Foresterhill, Aberdeen, UK; 3 Department of Cardiology, Imperial College Healthcare NHS Trust, London, UK; 4 University of Birmingham & University Hospitals Birmingham NHS Foundation Trust, Birmingham, UK; 5 Hampshire Hospitals NHS Foundation Trust, Hampshire, UK; 6 Cambridge University Hospitals NHS Foundation Trust, Cambridge, UK; 7 University Hospital Southampton NHS Foundation Trust, Southampton, UK; 8 Guy’s and St Thomas’ NHS Foundation Trust, London, UK; 9 Newcastle Upon Tyne Hospitals NHS Foundation Trust, Newcastle, UK; 10 Edinburgh Clinical Trials Unit, Usher Institute, University of Edinburgh, Edinburgh, UK

## Abstract

**Objectives:**

To investigate whether the use of graduated compression stockings (GCS) offers any adjuvant benefit when pharmaco-thromboprophylaxis is used for venous thromboembolism prophylaxis in patients undergoing elective surgery.

**Design:**

Open, multicentre, randomised, controlled, non-inferiority trial.

**Setting:**

Seven National Health Service tertiary hospitals in the United Kingdom.

**Participants:**

1905 elective surgical inpatients (≥18 years) assessed as being at moderate or high risk of venous thromboembolism were eligible and consented to participate.

**Intervention:**

Participants were randomly assigned (1:1) to receive low molecular weight heparin (LMWH) pharmaco-thromboprophylaxis alone or LMWH pharmaco-thromboprophylaxis and GCS.

**Outcome measures:**

The primary outcome was imaging confirmed lower limb deep vein thrombosis with or without symptoms, or pulmonary embolism with symptoms within 90 days of surgery. Secondary outcome measures were quality of life, compliance with stockings and LMWH, lower limb complications related to GCS, bleeding complications, adverse reactions to LMWH, and all cause mortality.

**Results:**

Between May 2016 and January 2019, 1905 participants were randomised. 1858 were included in the intention to treat analysis (17 were identified as ineligible after randomisation and 30 did not undergo surgery). A primary outcome event occurred in 16 of 937 (1.7%) patients in the LMWH alone group compared with 13 of 921 (1.4%) in the LMWH and GCS group. The risk difference between the two groups was 0.30% (95% confidence interval −0.65% to 1.26%). Because the 95% confidence interval did not cross the non-inferiority margin of 3.5% (P<0.001 for non-inferiority), LMWH alone was confirmed to be non-inferior.

**Conclusions:**

For patients who have elective surgery and are at moderate or high risk of venous thromboembolism, administration of pharmaco-thromboprophylaxis alone is non-inferior to a combination of pharmaco-thromboprophylaxis and GCS. These findings indicate that GCS might be unnecessary in most patients undergoing elective surgery.

**Trial registration:**

ISRCTN13911492 .

**Figure:**
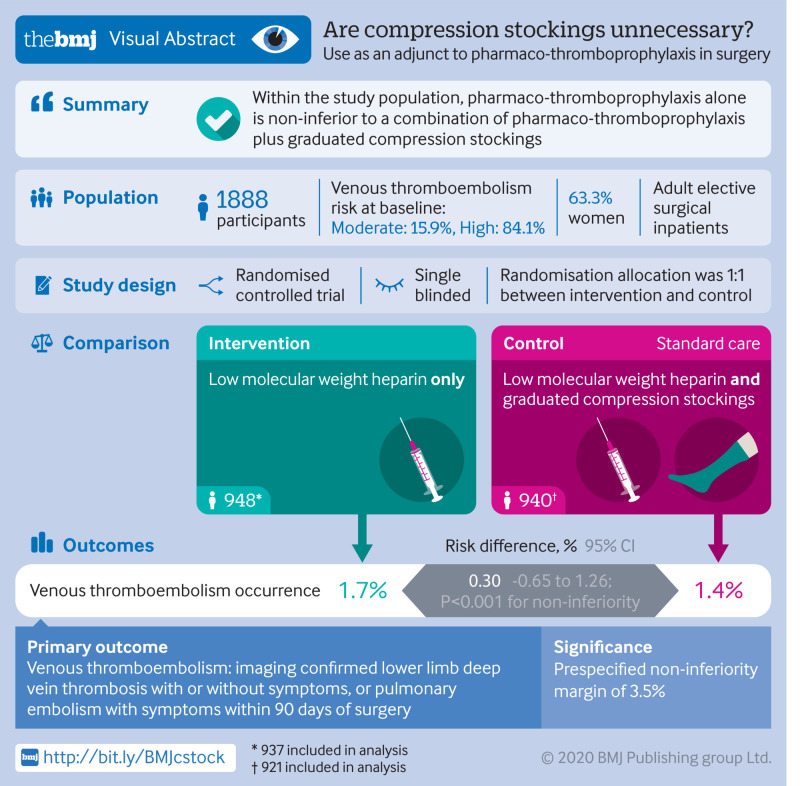


## Introduction

Venous thromboembolism (VTE) is a major global health problem and an important consideration for patients and clinicians at the time of elective surgery. Before elective surgical procedures, patients undergo a VTE risk assessment to guide the use of mechanical and pharmaco-thromboprophylaxis measures. Evidence exists for the use of pharmaco-thromboprophylaxis and graduated compression stockings (GCS) as mechanical thromboprophylaxis in this patient group.^1^ A systematic review and meta-analysis of 20 trials in surgical patients demonstrated much lower rates of deep vein thrombosis in patients randomised to GCS (134/1365, 9.8%) compared with those in control groups (282/1328, 21.2%)^2^; however, 19 of these trials were conducted before the year 2000. Rates of VTE have fallen over the past 50 years, partly because of thromboprophylaxis but also owing to changes in clinical practice.^3^ Therefore, the effectiveness of GCS in modern practice is uncertain. In surgical patients, use of low molecular weight heparin (LMWH) reduces clinical VTE and VTE without symptoms.^4^ Current guidelines widely recommend VTE prevention in the absence of contraindications by using a combination of GCS and pharmaco-thromboprophylaxis for patients undergoing elective surgery who are at moderate or high risk of VTE. In the United Kingdom, the Department of Health VTE risk assessment tool is advocated (https://www.nice.org.uk/guidance/ng89/resources/department-of-health-vte-risk-assessment-tool-pdf-4787149213),^5^ whereas internationally, the Caprini risk assessment tool (https://venousdisease.com/caprini-dvt-risk-assessment/) is also used to determine patients’ risk of VTE.^6^ ^7^

The effectiveness of GCS in modern practice has been questioned.^8^ A large randomised controlled trial in patients with stroke showed increased adverse events with GCS without an accompanying benefit in VTE reduction.^9^ In the context of elective surgery, a systematic review^10^ identified only one study in patients undergoing orthopaedic surgery that evaluated the benefit of GCS in addition to pharmaco-thromboprophylaxis. This single study found no additional benefit in using GCS.^11^ The review concluded that quantitative comparison or drawing definitive conclusions was difficult.^10^

We performed the graduated compression as an adjunct to pharmaco-thromboprophylaxis in surgery (GAPS) study,^12^ a large, pragmatic, multicentre, clinical effectiveness, two arm, parallel group, randomised controlled non-inferiority trial. In this study we evaluated the addition of GCS to LMWH for VTE prevention in patients undergoing elective surgery. Our aim was to investigate whether thromboprophylactic dose LMWH alone was non-inferior to thromboprophylactic dose LMWH and GCS in preventing imaging confirmed lower limb deep vein thrombosis with or without symptoms, or pulmonary embolism with symptoms up to 90 days after surgery.

## Methods

### Study design

Between May 2016 and January 2019, we enrolled participants in this randomised, non-inferiority clinical trial at seven centres across the UK (supplementary appendix). Details of the trial design and implementation are provided in the protocol, which has been published previously.^12^ All participants provided written informed consent and a trial steering committee and independent data monitoring committee provided the study oversight.

### Participants

Patients aged 18 years and older who were undergoing elective surgery and were assessed as being at moderate risk (score 1) or high risk (score ≥2) of VTE using the Department of Health VTE risk assessment tool^5^ (supplementary figure, web appendix 3) were screened for eligibility. We also assessed the VTE risk by using the Caprini risk assessment tool to evaluate applicability worldwide.^7^ We excluded female patients if they were pregnant, and any patients if LMWH or GCS were contraindicated, if they had a thrombophilia or thrombotic disorder, if they required therapeutic anticoagulation, or if they had a history of VTE. We also excluded patients if they required inferior vena cava filter insertion, intermittent pneumatic compression beyond the post anaesthesia recovery area, extended thromboprophylaxis (beyond discharge), or if they needed a cast or brace to be applied in the operating theatre. All participants provided written informed consent.

### Changes to study design

Because of the low event rate (blinded analysis on 1294 participants) and after input from the trial steering committee and independent data monitoring committee, the study design was revised in December 2017 (full details in the study protocol and supplementary appendix) to stratify the recruitment by age (<65 or ≥65 years) and VTE risk (moderate or high). We abandoned the group sequential design and respecified appropriate non-inferiority for these four subpopulations. Findings are reported for the original unstratified sample and the revised stratified sample.

### Randomisation and masking

Participants were randomised 1:1 to either pharmaco-thromboprophylaxis using LMWH alone or LMWH and GCS before they had surgery. Randomisation was through a web based application hosted by the University of Aberdeen Centre for Healthcare Randomised Trials. We used a minimisation algorithm that incorporated centre, moderate or high risk of VTE (VTE risk was not included after December 2017 because all participants recruited after this date were at high risk) and sex; a random element was also incorporated. Participants and investigators were not masked to treatment allocation. Vascular scientists or technologists who performed the duplex ultrasound scans were blinded to the study allocation. Participants were asked to remove their stockings and not to disclose their treatment allocation to staff who performed the scan.

### Interventions

Participants in both study groups were given thromboprophylactic doses of LMWH for the period of hospital admission. The formulation of LMWH varied by centre, however dosing was the standard manufacturer’s thromboprophylactic dose for the formulation. Participants randomised to adjuvant GCS received above or below knee GCS (providing 18 mm Hg compression at the level of the ankle) in addition to LMWH. Participants were asked to wear GCS for the duration of hospital admission. The brand and length of GCS supplied was guided by local standard policies. Previous studies have shown below knee GCS to be comparable to full length GCS in preventing deep vein thrombosis.^13^ Participants randomised to LMWH alone were asked not to wear any kind of compression stocking for 90 days after surgery.

### Outcomes

The primary outcome was new VTE within 90 days of surgery, either lower limb deep vein thrombosis with or without symptoms, proven by duplex ultrasound, or pulmonary embolism with symptoms confirmed by imaging. Participants were invited to undergo a bilateral full lower limb duplex ultrasound scan between 14 and 21 days after surgery to capture the peak incidence of VTE.^14^ ^15^ Whole leg venous ultrasound scans were performed, which evaluated deep veins from the common femoral vein to calf veins. Symptoms suggestive of VTE could trigger imaging at any point during the trial. If a positive diagnosis was made, the treating clinical team and local principal investigator were informed and the patient was treated in accordance with local hospital guidelines. An anonymised copy of the scan report was sent to the trial coordinating centre where the primary outcome was verified by an independent blinded clinical expert. The 90 day endpoint is in line with the definition of hospital acquired thrombosis.^16^ ^17^ ^18^ Secondary outcome measures included quality of life (assessed by using EQ-5D-5L (EuroQol five dimensions five levels)), compliance with stockings and LMWH, GCS related lower limb complications, bleeding complications, adverse reactions to LMWH, and all cause mortality.

### Statistical analysis

The supplementary material gives full details of the original and revised sample size calculations. The original sample size was 2236 for the primary outcome of VTE at 90 days. This sample was intended to have 90% power at a one sided 2.5% level of significance to detect a 3.5% non-inferiority margin (considered to be clinically important) over an assumed 6% event rate in the group randomised to LMWH and GCS; this rate was adjusted for 10% loss to follow up and the group sequential design. We derived the imaging confirmed VTE event rate of 6% from a recent systematic review that identified randomised controlled trials with arms exploring VTE outcomes in patients who had elective surgery.^10^ This margin was chosen because clinicians would not tolerate more than 3.5% absolute deterioration in VTE event rate over combination therapy. The margin also preserves 61% of the established treatment effect over no intervention. When we stratified patients by age (<65 or ≥65 years) and VTE risk category (moderate or high risk), four subpopulations were identified.

The formal interim analyses in the group sequential design were specified in event time; that is, after several events had accumulated. After the trial steering committee identified an overall lower than anticipated event rate, the independent data monitoring committee were unable to perform their first planned analysis. Instead the blinded senior statistician performed a blinded analysis of the first 1294 randomised patients, which identified a group in which the event rate was higher. We recalculated the sample sizes for four subpopulations stratified by age and VTE risk, abandoning the group sequential design. For example, in the group of patients aged 65 years and older, who were assessed as being at high VTE risk, we needed 912 participants, which would allow detection of a 4.0% non-inferiority margin assuming a 3.6% control rate.

We analysed VTE risk within 90 days of surgery by generalised linear modelling adjusted for sex and we added a cluster robust error for centre. Both intention to treat and per protocol analyses were performed, which included only participants who received the intervention to which they were randomised. We used the upper limit of the two sided 95% confidence interval for the risk difference to infer non-inferiority. A non-inferiority P value was calculated. We performed sensitivity analyses by only including participants who had a duplex scan and separately including post-randomisation exclusions. Similar analyses were performed for the four subpopulations unless a zero event rate existed in each arm, when a one sided absolute incidence confidence interval was reported.

EQ-5D-5L data were analysed using a mixed effects repeated measures model adjusted for baseline score, VTE risk, sex, and a random effect for centre. We performed non-parametric bounds^19^ for the average causal effect for compliance with stockings and LMWH. Participants were classified as fully compliant with LMWH if they received all prescribed LMWH doses, and partially compliant when they received more than 50% of prescribed doses. Compliance with GCS was defined as good if participants wore stockings for at least 75% of their hospital admission. Because of the low number of events, no formal analyses were planned for GCS related complications, adverse reactions to LMWH, bleeding complications, and all cause mortality. The subpopulations were analysed in a similar way.

We performed a post hoc analysis of the Caprini score for the overall trial population by using a generalised linear model that adjusted for sex and we added a cluster robust error for centre. For our analysis, the Caprini score was categorised as low-high (score <5) and highest (score ≥5) owing to the small number of participants in some of the categories. We excluded participants from all analyses if they did not undergo surgery. All analyses were performed in Stata version 15.^20^

### Patient and public involvement

A member of the registered charity Thrombosis UK acted as patient representative, assisting from the grant writing stages through to study completion. She also sat on the trial steering committee, which made all the major decisions from the planning and design, to approving study amendments, and assisting with dissemination of the study results to study participants and the general public.

## Results

### Screening

**Fig 1:**
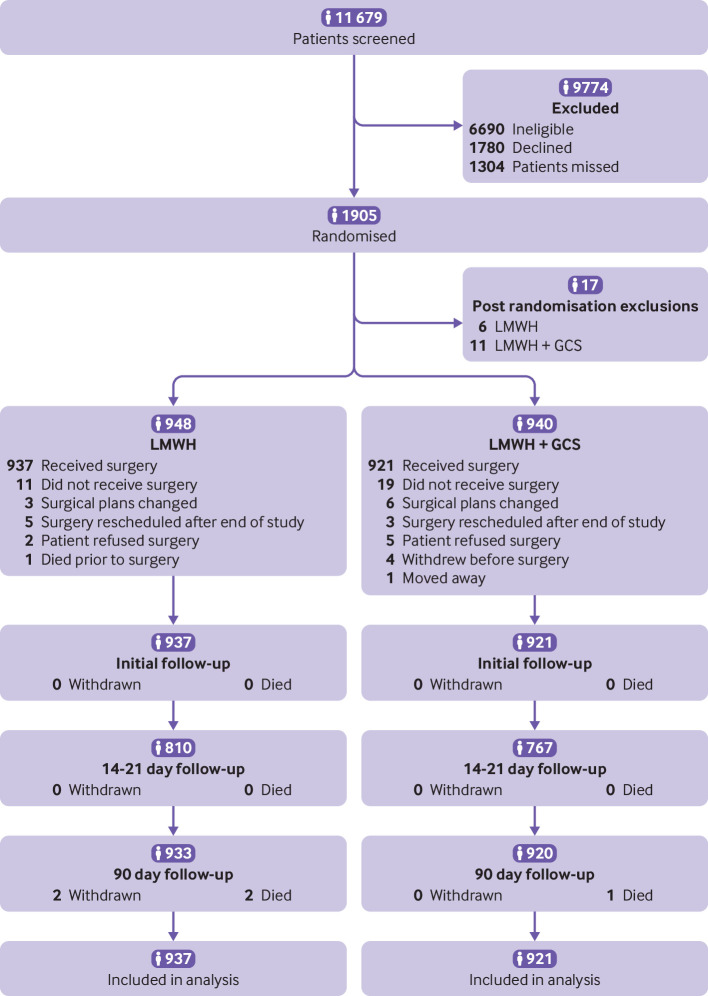
CONSORT (Consolidated Standards of Reporting Trials) diagram showing assessment of eligibility, enrolment, and follow-up

Of the 11 679 participants screened, 1905 were randomised with 17 classed as having post randomisation exclusions (fig 1 and table S2). Therefore, 1888 participants were included (948 in LMWH alone group and 940 in LMWH and GCS group). Of these, 1858 had surgery (937 in LMWH alone group and 921 in LMWH and GCS group) and were included in the intention to treat analysis. Table S3 presents details of participants in each subpopulation and table S1 gives reasons why participants were ineligible or declined to participate.

Baseline characteristics were similar in both groups (table 1 and table S4) and in the four subpopulations (table S5). Elective surgery was performed in 937/948 (98.8%) participants randomised to LMWH alone and 921/940 (98.0%) randomised to LMWH and GCS (table S6). The most common surgical procedures were general (upper gastrointestinal), obstetrics and gynaecology, and general (lower gastrointestinal; table S6). The allocated intervention was delivered in 758/948 (80.0%) in the LMWH alone group and 750/940 (79.8%) in the LMWH and GCS group (tables S6 and S7). In the LMWH and GCS group, 892/940 (94.9%) participants received GCS, of which 854/892 (95.7%) were below knee (tables S6 and S7).

**Table 1:** Baseline characteristics of study participants in the overall population. Values are numbers (percentages) unless stated otherwise

Characteristic	LMWH (n=948)	LMWH+GCS (n=940)
Age (years) (mean (SD))	59.3 (15.2)	58.1 (14.9)
Sex		
Male	347 (36.6)	346 (36.8)
Female	601 (63.4)	594 (63.2)
Body mass index (mean (SD))	28.7 (5.9)	29.0 (6.1)
VTE risk		
Moderate (score 1)	151 (15.9)	150 (16.0)
High (score ≥2)	797 (84.1)	790 (84.0)
Bleeding risk		
No bleeding risk	918 (96.8)	911 (96.9)
One or more risk factors	30 (3.2)	29 (3.1)
Caprini risk		
Low (score 0-1)	4 (0.4)	5 (0.5)
Moderate (score 2)	23 (2.4)	28 (3.0)
High (score 3-4)	275 (29.0)	267 (28.4)
Highest (score ≥5)	646 (68.1)	640 (68.1)
EQ-5D-5L* (mean (SD))	0.825 (0.185)	0.817 (0.192)
EQ-5D VAS† (mean (SD))	76.9 (17.5)	77.0 (18.1)
Oral contraceptives (women only)		
Yes	16/601 (2.7)	24/594 (4.0)
No	584/601 (97.2)	570/594 (96.0)
Missing	1/601 (0.2)	0/594 (0)
Hormone replacement therapy (women only)		
Yes	35/601 (5.8)	39/594 (6.6)
No	565/601 (94.0)	555/594 (93.4)
Missing	1/601 (0.2)	0/594 (0)
History of malignancy	213 (22.5)	197 (21.0)

EQ-5D-5L=EuroQol five dimensions five levels; EQ-5D VAS=EuroQol five dimensions visual analogue scale; GCS=graduated compression stockings; LMWH=low molecular weight heparin.

No significant differences existed between trial groups.

* LMWH n=942; LMWH+GCS n=926.

† LMWH n=941; LMWH+GCS n=923.

### Primary outcome

**Fig 2:**
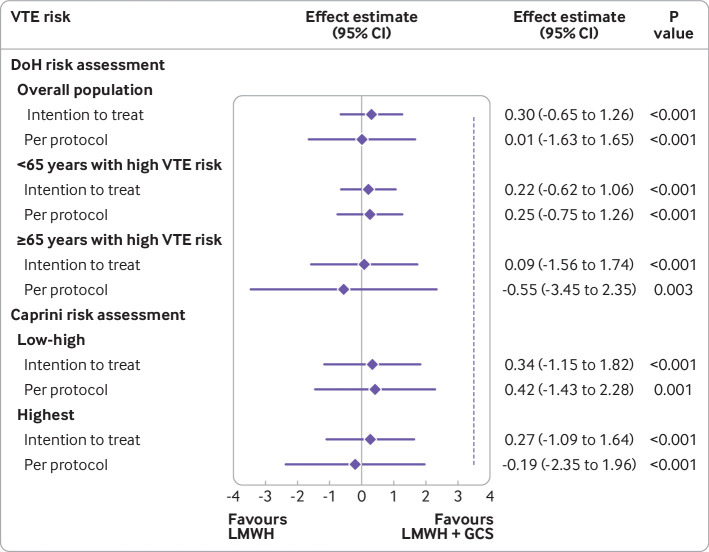
Venous thromboembolism (VTE) for overall population and subpopulations with high VTE risk (Department of Health (DoH) risk assessment tool^5^) and all populations (Caprini VTE risk assessment^7^). Data are effect estimates in percentages. Dashed vertical line is the non-inferiority margin (3.5%). GCS=graduated compression stockings; LMWH=low molecular weight heparin

In the prespecified intention to treat analysis, VTE occurred in 16/937 (1.7%) participants in the LMWH alone group compared with 13/921 (1.4%) in the LMWH and GCS group (risk difference 0.30%, 95% confidence interval −0.65% to 1.26%; P<0.001 for non-inferiority; fig 2). Because the 95% confidence interval did not cross the non-inferiority margin of 3.5%, the group randomised to LMWH alone was demonstrated to be non-inferior. Imaging confirmed pulmonary embolism occurred in 2/937 (0.2%) participants in the LMWH alone group compared with 1/921 (0.1%) in the LMWH and GCS group. Deep vein thrombosis with symptoms occurred in 2/937 (0.2%) participants in the LMWH alone group compared with 1/921 (0.1%) in the LMWH and GCS group. In patients who had full lower limb duplex imaging, deep vein thrombosis without symptoms was identified in 12/810 (1.5%) participants in the LMWH alone group compared with 11/767 (1.4%) in the LMWH and GCS group (table 2).

**Table 2:** Details on type of venous thromboembolism in overall population and subpopulations. Values are numbers (percentages) unless stated otherwise

Type of VTE	LMWH	LMWH+GCS
**Overall population (LMWH n=937, LMWH**+**GCS n=921)**		
VTE within 90 days	16 (1.7)	13 (1.4)
Type of VTE		
DVT with symptoms	2 (12.5)	1 (7.7)
DVT without symptoms identified by duplex	12 (75.0)	11 (84.6)
Imaging confirmed pulmonary embolism with symptoms	2 (12.5)	1 (7.7)
**Including exclusions after randomisation (LMWH n=943, LMWH**+**GCS n=932)**		
VTE within 90 days	16 (1.7)	13 (1.4)
Type of VTE		
DVT with symptoms	2 (12.5)	1 (7.7)
DVT without symptoms identified by duplex	12 (75.0)	11 (84.6)
Imaging confirmed pulmonary embolism with symptoms	2 (12.5)	1 (7.7)
**Including patients who had a duplex scan (LMWH n=810, LMWH**+**GCS n=767)**		
VTE within 90 days	16 (2.0)	13 (1.7)
Type of VTE		
DVT with symptoms	2 (12.5)	1 (7.7)
DVT without symptoms identified by duplex	12 (75.0)	11 (84.6)
Imaging confirmed pulmonary embolism with symptoms	2 (12.5)	1 (7.7)
**<65 years with high VTE risk (LMWH n=360, LMWH**+**GCS n=395)**		
VTE within 90 days	2 (0.6)	1 (0.3)
Type of VTE		
DVT with symptoms	0 (0)	1 (100.0)
DVT without symptoms identified by duplex	2 (100.0)	0

DVT=deep vein thrombosis; GCS=graduated compression stockings; LMWH=low molecular weight heparin; VTE=venous thromboembolism.

Results were similar in the per protocol analysis (LMWH alone 12/758 (1.6%), LMWH and GCS 12/750 (1.6%); fig 2) and the sensitivity analysis (table S8). Non-inferiority for LMWH alone was shown in all the subpopulations (fig 2 and table S9).

### Secondary outcomes

We found no differences in quality of life outcomes between groups at baseline, at one week or discharge from hospital, at 14-21 days, or at 90 days (table 3). Overall 750/940 (79.8%) participants randomised to LMWH and GCS had good compliance with stockings (table 3). Full compliance with LMWH was achieved in 768/948 (81.0%) in the LMWH alone group and in 755/940 (80.3%) in the LMWH and GCS group (table 3).

**Table 3:** Quality of life outcomes and compliance in overall population

Quality of life and compliance	LMWH (n=948)	LMWH+GCS (n=940)	Mean difference (95% CI)†	P value
**Received surgery (LMWH n=937, LMWH**+**GCS n=921)**				
EQ-5D-5L (No; mean (SD))				
Baseline	933; 0.825 (0.185)	910; 0.818 (0.192)	—	—
1 week or discharge	874; 0.648 (0.232)	839; 0.627 (0.244)	0.015 (−0.004 to 0.033)	0.12
14-21 days	846; 0.788 (0.202)	820; 0.773 (0.206)	0.011 (−0.008 to 0.030)	0.25
90 days	774; 0.856 (0.192)	743; 0.843 (0.197)	0.011 (−0.009 to 0.031)	0.27
EQ-5D VAS (No; mean (SD))				
Baseline	932; 77.0 (17.4)	907; 77.0 (18.2)	—	—
1 week or discharge	873; 68.2 (19.5)	837; 67.8 (20.1)	0.23 (−1.32 to 1.79)	0.77
14-21 days	846; 77.4 (17.4)	819; 77.2 (17.0)	−0.04 (−1.62 to 1.54)	0.96
90 days	773; 80.2 (17.9)	743; 80.7 (18.2)	−0.29 (−1.94 to 1.37)	0.73
Compliance with GCS* (No (%))	37 (4.0)	750 (81.4)	(−0.04 to 0.18)	—
Compliance with LMWH (No (%))				
Received all prescribed LMWH doses	768 (82.0)	755 (82.0)	(−0.81 to 0.19)	—
Received ≥50% of prescribed doses	779 (83.1)	762 (82.7)	(−0.82 to 0.18)	—

EQ-5D-5L=EuroQol five dimensions five levels; EQ-5D VAS=EuroQol five dimensions visual analogue scale; GCS=graduated compression stockings; LMWH=low molecular weight heparin.

* Stockings worn for 75% of total admission time.

† Non-parametric bounds for average causal effect for compliance outcome.

Table 4 summarises complications and all cause mortality by treatment received. GCS related complications were reported in 50/787 (6.4%) patients who received LMWH and GCS, and in 5/160 (3.1%) patients who received GCS only; most of these complications were discomfort. Adverse reactions to LMWH were reported in 6/779 (0.8%) patients who received LMWH alone and in 2/787 (0.3%) patients who received LMWH and GCS (table 4). Two (0.3%) deaths occurred in patients who received LMWH alone and in 1/132 (0.8%) patient who received no treatment; all deaths were unrelated to either LMWH or GCS. Serious adverse events (n=239) were reported in 210 participants; eight events were considered to be probably related to LMWH (table 4 and table S10).

**Table 4:** Complications and all cause mortality in overall population as treated. Values are numbers (percentages) or numbers

Complications and mortality	LMWH (n=779)	LMWH+GCS (n=787)	GCS only (n=160)	Neither (n=132)
GCS related complications*	2 (0.3)†	50 (6.4)	5 (3.1)	1 (0.8)†
Discomfort	2	41	4	1
Skin break or ulcer	0	1	0	0
Skin rash	0	4	0	0
Other	0	21	1	0
Adverse reactions to LMWH*	6 (0.8)	2 (0.3)	0	0
Abnormal liver enzyme	7	0	0	0
Other	2	2	0	0
Bleeding complications	5 (0.6)	4 (0.5)	1 (0.6)	0
All cause mortality	2 (0.3)	0	0	1 (0.8)

GCS=graduated compression stockings; LMWH=low molecular weight heparin.

A proportion of participants had treatment that was outside the treatment to which they were randomised (“GCS only” and “Neither” columns; “Neither” indicates that they received neither LMWH nor GCS).

* Participants could have more than one complication.

† Participants wore stockings for less than an hour, therefore classified as not wearing stockings.

When we used the Caprini score in place of the Department of Health VTE risk assessment in a post hoc analysis, 1286/1888 (68.1%) participants were classified at highest risk of VTE (score ≥5). On intention to treat analysis, for patients who had surgery, 14/640 (2.2%) VTE episodes occurred in the LMWH alone group compared with 12/625 (1.9%) in the LMWH and GCS group (risk difference 0.27%, 95% confidence interval −1.09% to 1.64%, P<0.001 for non-inferiority) (fig 2 and table S11).

## Discussion

### Principal findings

This multicentre, pragmatic randomised study showed that pharmaco-thromboprophylaxis with LMWH alone is non-inferior to pharmaco-thromboprophylaxis with LMWH and GCS in patients who had elective surgery and were assessed as being at moderate or high risk of VTE. This finding was sustained when we examined subpopulations based on age (<65 or ≥65 years) and baseline VTE risk (moderate or high).

### Strengths and weaknesses of the study

The case mix of elective surgical procedures included in this study is similar to the proportions undertaken nationally within the UK. Therefore, the results appear to be applicable to the wider elective surgical population. Most participants in this trial were deemed to be in the highest risk category for VTE, whether assessed using the Department of Health risk assessment tool^5^ or the Caprini score.^7^

We acknowledge that our study has limitations. Firstly, 281/1858 (15.1%) of participants did not receive a duplex ultrasound scan, which could have detected more patients with deep vein thrombosis without symptoms. However, the proportion was relatively small and numbers of missed scans were comparably distributed across the two randomised groups. The proportion that did not receive a duplex ultrasound scan would have been expected to contribute approximately one additional asymptomatic event to each treatment group, and therefore would probably not have influenced the overall findings. Secondly, the subgroup of participants aged 65 years and older assessed as being at moderate VTE risk was underrepresented in the study, which reflects that this group is underrepresented in the general population. In fact, 797/948 (84.1%) participants in the LMWH alone group and 790/940 (84.0%) in the LMWH and GCS group were assessed as being high risk (score ≥2) for VTE according to the Department of Health VTE risk assessment tool. Additionally, more than two thirds of participants were highest risk (score ≥5) when assessed by using the Caprini score, with non-inferiority of LMWH alone compared with LMWH and GCS in these highest risk populations.

### Comparison to other studies

The results of our study add to a growing body of evidence that does not support the use of GCS when pharmacological measures are not contraindicated and are given.^8^ ^21^ In our study population, 854/892 (95.7%) participants received below knee stockings, which probably reflects UK practice. This study cannot absolutely conclude that thigh length GCS have no benefit. However, in a randomised controlled trial of inpatients with acute stroke, thigh length GCS did not significantly reduce the risk of developing femoro-popliteal deep vein thrombosis with or without symptoms,^9^ and were associated with more skin complications. These findings resulted in a reduction in the use of GCS in patients with stroke.

The findings of this study are supported by data from a single centre where the policy has been not to use GCS in patients receiving elective surgery who are given a thromboprophylactic dose of LMWH. VTE outcomes reported by this centre are comparable to national figures.^22^

### Meaning of the study

A 15.4% reduction in 90 day VTE mortality has recently been reported since the introduction of a systematic approach to preventing hospital associated VTE in the UK in 2010.^23^ An opinion states that “we have entered an era in which the rates of VTE after surgery have fallen significantly through improved care and pharmaco-thromboprophylaxis such that combined thromboprophylaxis using LMWH and GCS might no longer be necessary.”^23^

It is notable that in this study event rates were much lower than expected based on previously published studies.^10^ These rates could be partly attributed to shorter lengths of stay, improved operative techniques, and analgesic regimens that allow earlier mobilisation. It is clear that current guidelines are largely based on historical data and should be revisited.

The findings of this study will probably have clinical implications on the administration of GCS in patients undergoing elective surgical procedures. Initial estimates of the annual cost of purchasing and applying GCS to surgical patients assessed as being at moderate or high risk of VTE exceeded £63m in England ($79m; €72m).^12^ A substantial reduction of these costs, scaled globally and on a recurring basis, has the potential to have a major positive financial impact on hospital healthcare systems.

In conclusion, this multicentre randomised trial showed that LMWH alone is non-inferior to LMWH and GCS for the prevention of VTE in patients undergoing elective surgery who are at moderate or high risk of VTE. Non-inferiority was shown across individual risk subpopulations. If we consider the potential adverse events and cost of GCS, urgent revision of national and international VTE prevention guidelines is recommended.

### Unanswered questions and future research

We excluded patients in whom current evidence supports pharmacological VTE prophylaxis after discharge from hospital,^1^ ^24^ such as those undergoing major joint replacement surgery or abdominopelvic cancer resections. This exclusion should be considered when translating and implementing the findings of this study into clinical practice. Further studies are needed to evaluate the adjuvant benefit of GCS in these groups of patients and in those undergoing surgery in the emergency setting.

**What is already known on this topic**

Venous thromboembolism prophylaxis is needed in most patients undergoing surgical procedures
The adjuvant benefit of graduated compression stockings in addition to pharmaco-thromboprophylaxis in these patients is unclear

**What this study adds**

This trial showed that in patients who had elective surgical procedures and were assessed to be at moderate or high risk of venous thromboembolism, pharmaco-thromboprophylaxis alone is non-inferior to pharmaco-thromboprophylaxis and graduated compression stockings
Current guidelines based on historical data should be revised

## Web extra

Extra material supplied by authors

**Web appendix 1:** GAPS supplementary tables and figures

**Web appendix 2:** Supplementary appendix—plain English summary submitted to the funders National Institute of Health Research (NIHR)

**Web appendix 3:** Supplementary figure—risk assessment for venous thromboembolism (VTE)

## Notes

Contributors: AHD, JS, CB, AB, KD, TE, MSG, ZH, BJH, DW, and GS were involved in study design, data collection, data interpretation, writing, critical review, and final approval of the manuscript. JN was involved in study design, data interpretation, writing, critical review, and final approval of the manuscript. RL was involved in the data collection, study coordination, writing, and critical review of the manuscript. JH performed the data analysis and was involved in data interpretation, writing, and critical review of the manuscript. The corresponding author attests that all listed authors meet authorship criteria and that no others meeting the criteria have been omitted. AHD acts as guarantor for the work. The corresponding author had full access to all the data in the study and had final responsibility for the decision to submit for publication.

Ethical approval: The study was approved by the UK National Research Ethics Service (London-City Road and Hampstead Committee on 8 February 2016, reference number 16/LO/0015).

Data sharing: Requests to access data should be addressed to a.h.davies@imperial.ac.uk. Deidentified individual participant data (including data dictionary) will be available to medical researchers by request in accordance with local registration and ethical approval when the article has been published until 1 November 2029. All proposals requesting data access will be reviewed on a case by case basis and will need to specify an analysis plan and will need approval of the scientific board before any data can be released.

The lead author affirms that this manuscript is an honest, accurate, and transparent account of the study being reported; that no important aspects of the study have been omitted; and that any discrepancies from the study as planned (and, if relevant, registered) have been explained.

Dissemination to participants and related patient and public communities: A newsletter describing the results of the trial and thanking participants for their contribution to the study will be sent to all participants and public communities who requested to be informed of the results.
