# Supplementary material for: Graduated compression stockings as adjuvant to pharmaco-thromboprophylaxis in elective surgical patients (GAPS study): randomised controlled trial
Source: BMJ. 2020 May 13;369:m1309. doi: 10.1136/bmj.m1309 (PMC7219517; doi:10.1136/bmj.m1309)
Supplement: Supplementary file 1 — Web appendix 1: GAPS supplementary tables and figures [file shaj053785.ww1.pdf]

GAPS Supplementary Tables and Figures

Acknowledgements

Supplementary Statistical Appendix

Table S1. Ineligible and declined reasons

Table S2. Reasons for post-randomisation exclusions

Table S3. Subpopulations

Table S4. Baseline characteristics – overall population[1]

Table S5. Baseline characteristics – sub populations

Table S6. Surgery details and treatment received – overall population

Table S7. Surgery details and treatment received – sub-populations

Table S8. Confirmed VTE within 90 days – sensitivity analyses for overall population

Table S9. Confirmed VTE within 90 days

Table S10. Serious adverse events – overall population by as treated

Table S11. Caprini subpopulations

Acknowledgements

The GAPS team would like to thank the NHS trusts and participating principal investigators and their colleagues for recruiting and monitoring trial participants. These include (in alphabetical order of participating hospitals followed by the local principal investigators and their colleagues):

Mr Manjit S Gohel, Simone Evans, Aileen Nacorda, Andrea Pentelow, Rebecca Rastall, Debbie Read, Umar Sadat, Robyn Weston (Cambridge University Hospitals NHS Foundation Trust)

Professor Beverley J Hunt, Linda Debattista, Johanna Young, Anisur Rahman, Karen Breen (Guy's and St Thomas' NHS Foundation Trust)

Professor Andrew Bradbury, Lisa Kelly, Gareth Bate, Huw Davies, Matt Popplewell, Martin Claridge, Sam Stafford, Athanasia Filippa, Rachel O'Beney, Kaytie Webley, Miriam Sangombe, Julie Markley, Lewis Meecham, Charles Hendrickse, Paul Super, Markos Daskalakis, Olga Tucker, Rishi Singhal, Gamal Barsoum, Balapathiran Balasubramanian, Anwar Haq, John Canny, Harmeet Khaira, Michael Foster, Shankar Chandrasekharan, Bhupendra Sarmah, Ramasamy Jaganathan, Ather Abdelbaky, Josaiah Adenugba, Odunayo Kalejaiye, Stewart Phillips, Christopher Dowson, Salena Bains, Hynek Mergental, Vasileios Charalampakis, Asif Sheikh, Javeria Iqbal, Kuo Ho. Neil Smith, Ivan Kalik, Katy Bloom, Xian Shen, Chloe Rai, Tracy Ellis (University Hospitals Birmingham NHS Foundation Trust)

Alun Davies, Joseph Shalhoub, Vernisha Ali, Karen Dhillon, Tuong-Vi Le Magowan, Layla Bolton Sagdhaoui, Francine Heatley, Laura Burgess, Snezhina Tsvetkova, Urmila Phuyal, Aaron Clarke, Mary Ellis, Janice Sweid, Mohammed Aslam, Tara Harvey, Patrick Daly, Chandanjit Kainth, Janice Okamgba (Imperial College Healthcare, NHS Trust)

Mr Simon Toh, Mr Nicholas Carter, Sheeba Babu, Elizabeth Hawes, Tracey Lee, Zoe Daly, Katherine Ellinor, Alison Blythin, Claire James, Amanda Hungate, Stacey Valentine, Yasmin Harrington-Davies, Deirdre Rogers, Kerry Gunner, Kerrie Scott, Johanna Mouland, Arlene Lee, Maria Moon, Ann Holmes, Rebecca Doel, Rebecca Baker, Alison Charig, Carol Martin, Carole Harris, Julie Kennedy, Penny Gill, Catherine Rogan, Ming Yeung, Sophie McDermott, Rachel Deadman (Portsmouth Hospitals NHS Trust)

Dr Tamara Everington, Dr Jonathan Cullis, Vicky King, Caroline Clarke, Jenny Brown, Lehentha Mattocks, Nicole Tipler, Linda Harris, Siju Matthew, Michele Tribbeck, Emma Stobie, Susan Richards, Susan Challis (Salisbury District Hospital)

Professor David Warwick, Mr Zaed Hamady, Mcdonald Mupudzi, Marie Nelson, Kirsty Gladas, Margarida Rodriguez, Andrew Guy, Amy Long, Danielle McCraken, Rachel Schranz,Lucy Johnstone, Michele Jordan, Emma Munro, Abbie Morley, Debbie Spiegal, Anne Daw, Jasmina Mandair, Lucy Gledhill- Gilvarry, Shery Michael, Jo Stanley, Priya Mathew, Jana Rojkova, Esther Pyle, Puja Patel, Carol Collins, Joel Ansell, Carol Harris (University Hospital Southampton NHS Foundation Trust)

Mr. Tristan Lane for the core lab data work.

Centre for Healthcare Randomised Trials (CHaRT) Aberdeen

The following members were part of the wider GAPS study team: Ms. Francine Heatley, Trial Manager; Mrs. Alison MacDonald, database team; Mr. Mark Forrest, Mr. Brian Taylor, Dr. Samantha Wileman, Quality assurance.

Patient and public involvement
Annya Stephens-Boal was involved in the original design during the grant application stages and was an active member of the Trial Steering Committee throughout the study.

GAPS study Heath Technology Assessment National Institute of Healthcare Research grant applicants: Professor Alun H Davies, Mr. Joseph Shalhoub, Professor Beverley J Hunt, Professor Gerry Stansby, Dr. Tamara Everington, Dr. Christopher Baker, Professor Andrew Bradbury, Mr. Manjit S Gohel, Professor John Norrie, Sister Karen Dhillon, Ms. Annya Stephens-Boal, and Professor David Warwick

Trial Management Group
The Trial Management Group comprised Professor Alun Davies (as chief investigator), Ms. Rebecca Lawton (as trial manager), Mrs. Alison MacDonald (as senior trials manager), Mr. Mark Forrest (as programming manager) Professor John Norrie (as senior statistician), Ms. Jemma Hudson (as statistician), and Mr. Joseph Shalhoub (as co-applicant providing clinical input).

We would also like to thank members of our two oversight committees; Trial Steering Committee: Professor Robert Hinchliffe (Chair, Clinical Professor of Vascular Surgery); Dr. Peter MacCallum (Senior Lecturer in Haematology); Mr. Nick Hickey (Consultant Vascular Surgeon); Dr Stephen Gerry (Medical Statistician and NIHR Doctoral Research Fellow) and Ms. Annya Stephens-Boal (lay member, Thrombosis UK) who provided invaluable input and advice as the independent lay member over the course of the study; Data Monitoring Committee; Mr. Richard Bulbulia (Chair, Consultant Vascular Surgeon); Dr. Adam Rye (Consultant Haematologist); Professor Jonathan Emberson (Associate Professor, Medical Statistics and Epidemiology) for their support and guidance.

Supplementary Statistical Appendix

Changes to the study design – rationale and details

The formal interim analyses in the group sequential design were specified in event time after a certain number of events had accumulated, with the first interim analysis scheduled at 25% of these events. It became apparent during the trial that the observed event rate was lower than assumed and not enough for the iDMC to formally reach the first interim analysis. Following an analysis on 1294 participants randomised who had achieved 90 day follow up, the group sequential design was revised in December 2017 due to the observed low event rate. Details of this change are detailed in the study protocol[25]. In brief, only looking at blinded data (aggregated across the two randomised groups i.e. with no consideration of any emerging treatment effect), it was discovered that the risk of a primary outcome was clearly heavily stratified by age and VTE risk. In aggregate we would have expected around 80 events if the two interventions were the same, but we had observed just 16 VTE events at 90 days. There were no events observed in those aged <65 years assessed as being at moderate VTE risk, very few events observed in those <65 years assessed as being at high VTE risk, and nearly all the observed events occurred in those ≥65 years at high VTE risk. The remaining subpopulation of those aged ≥65 years with moderate risk was virtually empty. It was realised that this stratification meant, in practice, we had three separate randomised trials, two of which had already achieved sufficient recruitment to be adequately powered for clinically meaningful non-inferiority hypotheses (the <65 years, at moderate VTE risk and the <65 years, at high VTE risk). So from January 2018 further recruitment was restricted to individuals aged ≥65 years assessed as being at high VTE risk, which needed to achieve a sample size of around 900 to be adequately powered for a new non-inferiority hypothesis. At the same time the group sequential design was abandoned, since we were two-thirds recruited to this new target, and replaced with a single analysis at study end. Statistically, having three trials all adequately powered with fewer participants overall (~1900) than the original trial (~2300) exploits the reduction in variability as the binomial proportion reduces.

Original and revised sample sizes

Original sample size. The original sample size assumed a primary outcome of VTE at 90 days for 6% on standard care (pharmacothromboprophylaxis and GCS). The imaging-confirmed VTE event rate was derived

from a recent systematic review which identified RCTs with study groups exploring VTE outcomes in elective surgical patients[10]. Assuming a non-inferiority margin of 3.5% (considered to be clinically important) and a 1-sided test at 2.5% level of significance and 90% power, the study needed a fixed sample size of 1936 participants. Adjusting for 10% loss to follow up, and a group sequential design with interim analyses for efficacy at 25%, 50%, 75% and 100%, and one interim analysis for futility at 50% of expected events inflates this maximum sample size to 2236. Historically, without pharmacology or stockings, the untreated event rate was at least 15%, so the 3.5% non-inferiority margin preserves just over 60% of the established benefit of standard care.

Revised sample size. After the blinded analysis of the first 1294 randomised with 90 day follow up, it was clear that there were four distinct subpopulations stratified by age and VTE risk score (see above), rather than a single homogenous population. We reworked the sample sizes within these four strata, observing that for two of the strata we already had recruited an adequate sample size (the two strata for those aged <65 years), for a third (aged ≥65 years assessed as moderate VTE risk) it was futile to continue due to the very small membership of this strata, and that we still only needed to recruit further to the fourth strata (aged ≥65 years assessed as high VTE risk) from December 2017 onwards. At 90% power and the 1-sided 2.5% level of significance, with no adjustment for multiple comparisons, the new sample sizes (n) required for assumed event rate (e) and non-inferiority margins (Δ) were: subpopulation 1: age <65 years, moderate VTE risk (n=258, e=0.1%, Δ=1.3%); subpopulation 2: age <65 years high VTE risk (n=733, e=0.6%, Δ=1.85%); subpopulation 3: age ≥65 years moderate VTE risk – not feasible; subpopulation 4: age ≥65 years high VTE risk n=912 e=3.6%, Δ=4.0%). These revised sample size calculations adjust for 10% loss to follow up, but not the group sequential design as this was abandoned at this stage, with no alpha spent. The determination of these new non-inferiority margins was not based on the existing literature, given that the analysis of the first 1274 participants of this trial had demonstrated clearly that the literature was not accurately informative about the cohort that was being recruited contemporaneously. Instead, it was largely based on (a) what the research group felt would apply as an appropriate non-inferiority margin given the original research to establish a non-inferiority margin of 3.5% in a population experiencing 6% event rate on drugs and stockings; and (b) what was feasible to recruit in the single remaining strata that was experiencing nearly all the observed events, given the time and funding constraints of a publicly funded study that had already experienced delays in reaching the point of re-design.

Table S1. Ineligible and declined reasons

	N
Ineligible[1]	N=6690
Day case	2427
Patients requiring thromboprophylaxis to be extended beyond discharge	1270
Patients having intermittent pneumatic compression (IPC) beyond theatre and recovery	682
Contraindications to low molecular weight heparin (LMWH)	679
Individuals requiring therapeutic anticoagulation	643
Contraindications to GCS	345
Previous venous thromboembolism	331
Lack of capacity	290
Not 65 and Not high risk on VTE[2]	244
Clinical team did not give agreement	168
Surgery cancelled	134
Documented or known thrombophilia or thrombogenic disorder	110
Contraindications to low molecular weight heparin (LMWH)- high risk Bleeding	21
Age < 18	14
Pregnancy	7
Application of a cast or brace in theatre	7
Patients requiring inferior vena cava (IVC) filter	6
Unknown	23
Declined	N=1780
No reason given	784
Attending clinic	567
Participant wanted stockings	289
Not interested	92
Family declined	25
Does not want stockings	18
Does not want LMWH	5

Values are numbers. [1]more than one reason is possible. [2]Exclusion criteria post December 2018 when recruitment did not include these participants.

Table S2. Reasons for post-randomisation exclusions

	**LMWH N=6**	**LMWH + GCS N=11**
To receive extended thromboprophylaxis	4	6
Peripheral vascular disease	0	2
Already on anticoagulant	1	1
Not for LMWH	0	1
Thrombotic disorder	0	1
Reclassified to low risk of VTE	1	0

Values are numbers

Table S3. Subpopulations

	LMWH N=948	LMWH + GCS N=940	Total N=1888
<65 years with moderate VTE risk	139 (14.7)	141 (15.0)	280 (14.8)
<65 years with high VTE risk	362 (38.2)	403 (42.9)	765 (40.5)
≥65 years with moderate VTE risk	12 (1.3)	9 (1.0)	21 (1.1)
≥65 years with high VTE risk	435 (45.9)	387 (41.2)	822 (43.6)

Values are numbers (percent)

Table S4. Baseline characteristics – overall population[1]

	LMWH N=948	LMWH + GCS N=940
Ethnicity		
White British	811 (85.5)	817 (86.9)
White Irish	14 (1.5)	14 (1.5)
White Other	35 (3.7)	27 (2.9)
White & Black Caribbean	1 (0.1)	2 (0.2)
White & Black African	0 (0)	2 (0.2)
White Asian	3 (0.3)	1 (0.1)
Other mixed background	1 (0.1)	0 (0)
Indian	15 (1.6)	6 (0.6)
Pakistani	15 (1.6)	9 (1.0)
Bangladeshi	1 (0.1)	2 (0.2)
Other Asian background	6 (0.6)	5 (0.5)
Caribbean	10 (1.1)	17 (1.8)
African	13 (1.4)	15 (1.6)
Black Other	5 (0.5)	1 (0.1)
Chinese	2 (0.2)	4 (0.4)
Other	16 (1.7)	18 (1.9)
Smoker		
Never	476 (50.2)	465 (49.5)
Ex-smoker	56 (5.9)	58 (6.2)
Ex-smoker <1 year	25 (2.6)	35 (3.7)
Ex-smoker <5 year	52 (5.5)	28 (3.0)
Ex-smoker >5 year	222 (23.4)	237 (25.2)
Current smoker	117 (12.3)	117 (12.4)
Alcohol consumption		
Never	238 (25.1)	217 (23.1)
Ex-drinker	144 (15.2)	132 (14.0)
Current drinker	566 (59.7)	591 (62.9)
Diet		
Vegetarian	47 (5.0)	39 (4.1)
Low meat diet	630 (66.5)	633 (67.3)
High meat diet (> 90g day)	271 (28.6)	268 (28.5)
Physical activity level		
Low	294 (31.0)	305 (32.4)
Moderate	562 (59.3)	544 (57.9)
Vigorous	92 (9.7)	91 (9.7)
Occupation		
Worker	133 (14.0)	136 (14.5)
Employee	211 (22.3)	221 (23.5)
Self-employed	63 (6.6)	66 (7.0)
Contractor	8 (0.8)	5 (0.5)
Director	8 (0.8)	6 (0.6)
Office holder	1 (0.1)	1 (0.1)
Unemployed	62 (6.5)	77 (8.2)
Student	4 (0.4)	5 (0.5)
Retired	458 (48.3)	423 (45.0)
Medication		
Anti-inflammatory	70 (7.4)	87 (9.3)
Statins	207 (21.8)	185 (19.7)
Antiplatelet therapy		
None	894 (94.3)	885 (94.1)
Single	52 (5.5)	53 (5.6)
Dual	1 (0.1)	2 (0.2)
Triple	1 (0.1)	0 (0)
Past surgical history	809 (85.3)	802 (85.3)
Past medical history[2]		
Previous myocardial infarction	10 (1.1)	15 (1.6)

Previous stroke	5 (0.5)	9 (1.0)
Treated hypertension	270 (28.5)	257 (27.3)
Other medical history	488 (51.5)	503 (53.5)
No past medical history	324 (34.2)	321 (34.1)
Previous pregnancies		
Yes	485 (51.2)	475 (50.5)
No	461 (48.6)	464 (49.4)
Missing	2 (0.2)	1 (0.1)

Values are n (%).[1] There were no significant differences between the trial groups. [2]Participants could have more than one past medical history

Table S5. Baseline characteristics – sub populations

	LMWH	LMWH + GCS
**<65 years with moderate VTE risk**	**N=139**	**N=141**
Age - n; mean (SD)	139; 43.8 (10.7)	141; 44.0 (10.7)
Gender		
Male	43 (30.9)	51 (36.2)
Female	96 (69.1)	90 (63.8)
CAPRINI risk		
Low (score 0-1)	3 (2.2)	5 (3.5)
Moderate (score 2)	16 (11.5)	19 (13.5)
High (score 3-4)	97 (69.8)	81 (57.4)
Highest (score ≥5)	23 (16.5)	36 (25.5)
EQ-5D-5L - n; mean (SD)	139; 0.861 (0.163)	140; 0.855 (0.177)
EQ-5D VAS - n; mean (SD)	139; 79.7 (17.9)	140; 80.3 (17.6)
Smoker		
Never	71 (51.1)	75 (53.2)
Ex-smoker	5 (3.6)	9 (6.4)
Ex-smoker <1 year	6 (4.3)	9 (6.4)
Ex-smoker <5 year	11 (7.9)	5 (3.5)
Ex-smoker >5 year	25 (18.0)	15 (10.6)
Current smoker	21 (15.1)	28 (19.9)
Anti-inflammatory	10 (7.2)	9 (6.4)
Antiplatelet therapy		
None	139 (100.0)	140 (99.3)
Single	0 (0)	1 (0.7)
**<65 years with high VTE risk**	**N=362**	**N=403**
Age - n; mean (SD)	362; 49.3 (11.0)	403; 49.4 (10.6)
Gender		
Male	108 (29.8)	107 (26.6)
Female	254 (70.2)	296 (73.4)
CAPRINI risk		
Low (score 0-1)	1 (0.3)	0 (0)
Moderate (score 2)	4 (1.1)	9 (2.2)
High (score 3-4)	126 (34.8)	147 (36.5)
Highest (score ≥5)	231 (63.8)	247 (61.3)
EQ-5D-5L - n; mean (SD)	361; 0.822 (0.189)	397; 0.803 (0.201)
EQ-5D VAS - n; mean (SD)	360; 76.3 (17.7)	396; 75.0 (19.5)
Smoker		
Never	191 (52.8)	207 (51.4)
Ex-smoker	28 (7.7)	27 (6.7)
Ex-smoker <1 year	11 (3.0)	13 (3.2)
Ex-smoker <5 year	24 (6.6)	15 (3.7)
Ex-smoker >5 year	44 (12.2)	78 (19.4)
Current smoker	64 (17.7)	63 (15.6)
Anti-inflammatory	30 (8.3)	42 (10.4)
Antiplatelet therapy		
None	351 (97.0)	393 (97.5)
Single	11 (3.0)	9 (2.2)
Dual	0 (0)	1 (0.2)
**≥65 years with moderate VTE risk**	**N=12**	**N=9**
Age - n; mean (SD)	12; 74.6 (5.2)	9; 71.3 (6.7)
Gender		
Male	7 (58.3)	7 (77.8)
Female	5 (41.7)	2 (22.2)
CAPRINI risk		
High (score 3-4)	8 (66.7)	3 (33.3)
Highest (score ≥5)	4 (33.3)	6 (66.7)
EQ-5D-5L - n; mean (SD)	12; 0.833 (0.227)	9; 0.885 (0.115)
EQ-5D VAS - n; mean (SD)	12; 76.7 (19.9)	9; 78.9 (13.6)
Anti-inflammatory	1 (8.3)	0 (0)

Antiplatelet therapy		
None	12 (100.0)	7 (77.8)
Single	0 (0)	2 (22.2)
**≥65 years with high VTE risk**	**N=435**	**N=387**
Age - n; mean (SD)	435; 72.3 (5.3)	387; 72.0 (5.2)
Gender		
Male	189 (43.4)	181 (46.8)
Female	246 (56.6)	206 (53.2)
CAPRINI risk		
Moderate (score 2)	3 (0.7)	0 (0)
High (score 3-4)	44 (10.1)	36 (9.3)
Highest (score ≥5)	388 (89.2)	351 (90.7)
EQ-5D-5L - n; mean (SD)	430; 0.816 (0.187)	380; 0.816 (0.187)
EQ-5D VAS - n; mean (SD)	430; 76.6 (17.2)	378; 77.8 (16.7)
Smoker		
Never	208 (47.8)	179 (46.3)
Ex-smoker	23 (5.3)	22 (5.7)
Ex-smoker <1 year	8 (1.8)	13 (3.4)
Ex-smoker <5 year	16 (3.7)	8 (2.1)
Ex-smoker >5 year	149 (34.3)	140 (36.2)
Current smoker	31 (7.1)	25 (6.5)
Anti-inflammatory	29 (6.7)	36 (9.3)
Antiplatelet therapy		
None	392 (90.1)	345 (89.1)
Single	41 (9.4)	41 (10.6)
Dual	1 (0.2)	1 (0.3)
Triple	1 (0.2)	0 (0)

Values are numbers (percent) unless otherwise stated.

Table S6. Surgery details and treatment received – overall population

	LMWH N=948	LMWH + GCS N=940
Did not received surgery	11 (1.2)	19 (2.0)
Reasons for not receiving surgery		
Surgical plans changed, not for surgery	3 (27.3)	6 (31.6)
Surgery rescheduled after the end of the trial	5 (45.5)	3 (15.8)
Patient refused surgery	2 (18.2)	5 (26.3)
Withdrew prior to surgery	0 (0)	4 (21.1)
Moved away	0 (0)	1 (5.3)
Died prior to surgery	1 (9.1)	0 (0)
Received surgery	N=937	N=921
Anaesthetic used		
General	914 (97.5)	899 (97.6)
Regional	15 (1.6)	19 (2.1)
Both	8 (0.9)	3 (0.3)
Details of treatment received		
LMWH + GCS	37 (3.9)	750 (81.4)
LMWH only	758 (80.9)	21 (2.3)
GCS only	18 (1.9)	142 (15.4)
Neither LMWH or GCS	124 (13.2)	8 (0.9)
Reasons for LMWH not given	N=142	N=150
Patient discharged early	54 (38.0)	58 (38.7)
Not prescribed	48 (33.8)	50 (33.3)
Clinical	25 (17.6)	25 (16.7)
Missed	4 (2.8)	6 (4.0)
No reason	8 (5.6)	7 (4.7)
Patient declined	2 (1.4)	1 (0.7)
Procedure abandoned in theatre	1 (0.7)	2 (1.3)
Other	0 (0)	1 (0.7)
Type of GCS	N=55	N=892
Above the knee	2 (3.6)	38 (4.3)
Below the knee	5 (9.1)	854 (95.7)
Not recorded	48 (87.3)	0 (0)
Surgical Procedure		
General – Upper gastrointestinal	293 (31.3)	289 (31.4)
Obstetrics and gynaecology	160 (17.1)	163 (17.7)
General – Lower gastrointestinal	106 (11.3)	116 (12.6)
Urology	86 (9.2)	79 (8.6)
General	50 (5.3)	54 (5.9)
General – Breast	54 (5.8)	50 (5.4)
Ear, nose, and throat	44 (4.7)	43 (4.7)
Neurosurgery	36 (3.8)	26 (2.8)
Plastics	18 (1.9)	21 (2.3)
Orthopaedics	11 (1.2)	17 (1.8)
Cardiothoracic	3 (0.3)	1 (0.1)
Vascular	2 (0.2)	1 (0.1)
Other	74 (7.9)	61 (6.6)

Values are numbers (percent)

Table S7. Surgery details and treatment received – sub-populations

	LMWH	LMWH + GCS
**<65 years with moderate VTE risk**	**N=139**	**N=141**
Received surgery	N=137	N=140
Treatment received		
Received allocated treatment	108 (78.8)	106 (75.7)
Details of treatment received		
LMWH + GCS	5 (3.6)	106 (75.7)
LMWH only	108 (78.8)	4 (2.9)
GCS only	4 (2.9)	30 (21.4)
Neither LMWH or GCS	20 (14.6)	0 (0)
Type of GCS	N=9	N=136
Above the knee	0 (0)	5 (3.7)
Below the knee	0 (0)	131 (96.3)
Not recorded	9 (100.0)	0 (0)
**<65 years with high VTE risk**	**N=362**	**N=403**
Received surgery	N=360	N=395
Treatment received		
Received allocated treatment	294 (81.7)	324 (82.0)
Details of treatment received		
LMWH + GCS	16 (4.4)	324 (82.0)
LMWH only	294 (81.7)	7 (1.8)
GCS only	5 (1.4)	60 (15.2)
Neither LMWH or GCS	45 (12.5)	4 (1.0)
Type of GCS	N=21	N=384
Above the knee	2 (9.5)	20 (5.2)
Below the knee	1 (4.8)	364 (94.8)
Not recorded	18 (85.7)	0 (0)
**≥65 years with moderate VTE risk**	**N=12**	**N=9**
Received surgery	N=11	N=9
Treatment received		
Received allocated treatment	9 (81.8)	9 (100.0)
Details of treatment received		
LMWH + GCS	0 (0)	9 (100.0)
LMWH only	9 (81.8)	0 (0)
Neither LMWH or GCS	2 (18.2)	0 (0)
Type of GCS	N=0	N=9
Below the knee	0 (0)	9 (100.0)
**≥65 years with high VTE risk**	**N=435**	**N=387**
Received surgery	N=429	N=377
Treatment received		
Received allocated treatment	347 (80.9)	311 (82.5)
Details of treatment received		
LMWH + GCS	16 (3.7)	311 (82.5)
LMWH only	347 (80.9)	10 (2.7)
GCS only	9 (2.1)	52 (13.8)
Neither LMWH or GCS	57 (13.3)	4 (1.1)
Type of GCS	N=25	N=363
Above the knee	0 (0)	13 (3.6)
Below the knee	4 (16.0)	350 (96.4)
Not recorded	21 (84.0)	0 (0)

Values are numbers (percent)

Table S8. Confirmed VTE within 90 days – sensitivity analyses for overall population

	LMWH	LMWH + GCS	RD	95% CI	p-value
Including post-randomisation exclusions	16/943 (1.7)	13/932 (1.4)	0.35%	(-0.72%, 1.43%)	<0.001
Including those that had a duplex scan	16/810 (2.0)	13/767 (1.7)	0.30%	(-0.95%, 1.54%)	<0.001

Values are n (%)

Table S9. Confirmed VTE within 90 days

	LMWH	LMWH + GCS	95% CI
**<65 years with moderate VTE risk**			
Intention-To-Treat	0/137 (0)	0/140 (0)	(0, 1%)
Per-protocol	0/108 (0)	0/106 (0)	(0, 1%)
**<65 years with high VTE risk**			
Intention-To-Treat	2/360 (0.6)	1/395 (0.3)	(-0.62%, 1.06%)
Per-protocol	2/294 (0.7)	1/324 (0.3)	(-0.75%, 1.26%)
**≥65 years with moderate VTE risk**			
Intention-To-Treat	0/11 (0)	0/9 (0)	(0, 14%)
Per-protocol	0/9 (0)	0/9 (0)	(0, 15%)

Values are numbers (percent). CI confidence interval. <65 years with moderate VTE risk and ≥65 years with moderate VTE risk was analysed using one-sided absolute incidence CI. For the other subgroups see Figure 2 in main paper for the analysis.

Table S10. Serious adverse events – overall population by as treated

	LMWH N=779	LMWH + GCS N=787	GCS only N=160	Neither N=132
Number of participants with an SAE	92 (11.8)	103 (13.1)	5 (3.1)	10 (7.6)
Total number of SAE's	110	112	5	12
Serious reason				
Death	2	0	0	1
Life-threatening	2	4	0	0
Required hospitalisation	72	74	3	11
Required prolonged hospitalisation	30	29	2	0
Resulted in persistent or significant disability	0	2	0	0
Other	5	4	0	0
Frequency				
Single Episode	87	92	5	9
Intermittent	4	4	0	2
Frequent	1	0	0	0
Continuous	16	14	0	1
Unknown	2	2	0	0
Severity				
Mild (aware of it easily tolerated)	21	13	1	0
Moderate (discomfort/interference with usual activity)	28	40	1	4
Severe (inability to carry out normal activity	53	51	3	7
Life threatening or disabling	8	8	0	1
Relationship to LMWH or GCS				
Not related	94	102	5	9
Unlikely	10	8	0	3
Possible	5	2	0	0
Probable	1	0	0	0
Primary SOC Term				
Gastrointestinal disorders	33	28	0	3
Injury, poisoning and procedural complications	25	20	1	2
Infections and infestations	15	13	0	2
Renal and urinary disorders	7	13	1	0
General disorders and administration site conditions	3	9	1	1
Respiratory, thoracic and mediastinal disorders	6	4	0	1
Surgical and medical procedures	3	4	1	0
Vascular disorders	4	2	0	2
Investigations	3	4	0	0

Cardiac disorders	1	4	0	1
Hepatobiliary disorders	1	3	0	0
Nervous system disorders	2	2	0	0
Neoplasms benign, malignant and unspecified (including cysts and polyps)	2	1	0	0
Reproductive system and breast disorders	1	2	0	0
Metabolism and nutrition disorders	0	2	0	0
Musculoskeletal and connective tissue disorders	1	1	0	0
Eye disorders	1	0	0	0
Product issues	1	0	0	0
Psychiatric disorders	1	0	0	0
Skin and subcutaneous tissue disorders	0	0	1	0

Values are numbers (percent) or numbers

Table S11. Caprini subpopulations

	LMWH	LMWH + GCS
**Low-High** (score 0-4)		
Intention-To-Treat	2/297 (0.7)	1/296 (0.3)
Per-protocol	2/236 (0.9)	1/238 (0.4)
**Highest** (score ≥5)		
Intention-To-Treat	14/640 (2.2)	12/625 (1.9)
Per-protocol	10/522 (1.9)	11/512 (2.23)

Values are numbers (%).
