# Supplementary material for: Graduated compression stockings as adjuvant to pharmaco-thromboprophylaxis in elective surgical patients (GAPS study): randomised controlled trial
Source: BMJ. 2020 May 13;369:m1309. doi: 10.1136/bmj.m1309 (PMC7219517; doi:10.1136/bmj.m1309)
Supplement: Supplementary file 2 — Web appendix 2: Supplementary appendix—plain English summary submitted to the funders National Institute of Health Research (NIHR) [file shaj053785.ww2.pdf]

Supplementary Appendix

Plain English Summary submitted to the funders National Institute of Health Research (NIHR), Health Technology Assessment (HTA), project number 14/140/61

Why did we conduct this research?

People attending hospital to undergo operations are at risk of developing blood clots in the legs known as a deep-vein thrombosis or DVT. These blood clots occur for several reasons, such as not being able to move around after an operation, changes occurring in the blood, or damage to the veins in which the blood travels.

To decrease the risk of getting a DVT, patients having operations are given tight elastic socks to wear called graduated compression stockings (GCS). They are also given medicine to thin the blood and stop it clotting.

There is not a lot of evidence to say that wearing elastic socks whilst in hospital will reduce the risk of developing a blood clot if a blood thinner is also given. Many patients say that the socks can hurt, cause bruising on the legs and can be difficult to put on.

The Graduated Compression as an adjunct to thromboprophylaxis in surgery (GAPS) trial investigated whether patients having an operation would benefit from wearing elastic socks as well as getting blood thinners, or if blood thinners on their own were enough to stop blood clots.

What did we do?

1905 patients having operations at seven hospitals in England agreed to take part in the trial. During their admission, half the patients were given elastic socks plus blood thinners, and the other half were given the blood thinners only.

What did we find?

We found that there was no significant difference in terms of the number of people who had a blood clot in either group of the trial. This could mean that the blood thinners alone are as good at stopping blood clots as blood thinners and elastic socks for patients having an operation.

What could be done next?

The NHS spends around £63 million per year across England on elastic stockings. This research may mean that some patients might not get any extra benefit from wearing them if they have already taken blood thinners.
