# Supplementary material for: Graduated compression stockings as adjuvant to pharmaco-thromboprophylaxis in elective surgical patients (GAPS study): randomised controlled trial
Source: BMJ. 2020 May 13;369:m1309. doi: 10.1136/bmj.m1309 (PMC7219517; doi:10.1136/bmj.m1309)
Supplement: Supplementary file 3 — Web appendix 3: Supplementary figure—risk assessment for venous thromboembolism (VTE) [file shaj053785.ww3.pdf]

RISK ASSESSMENT FOR VENOUS THROMBOEMBOLISM (VTE)

All patients should be risk assessed on admission to hospital. Patients should be reassessed within 24 hours of admission and whenever the clinical situation changes.

STEP ONE

Assess all patients admitted to hospital for level of mobility (tick one box). All surgical patients, and all medical patients with significantly reduced mobility, should be considered for further risk assessment.

STEP TWO

Review the patient-related factors shown on the assessment sheet against **thrombosis** risk, ticking each box that applies (more than one box can be ticked).

Any tick for thrombosis risk should prompt thromboprophylaxis according to NICE guidance.

The risk factors identified are not exhaustive. Clinicians may consider additional risks in individual patients and offer thromboprophylaxis as appropriate.

STEP THREE

Review the patient-related factors shown against **bleeding risk** and tick each box that applies (more than one box can be ticked).

Any tick should prompt clinical staff to consider if bleeding risk is sufficient
to preclude pharmacological intervention.

Guidance on thromboprophylaxis is available at:

National Institute for Health and Clinical Excellence (2010) Venous thromboembolism: reducing the risk of venous thromboembolism (deep vein thrombosis and pulmonary embolism) in patients admitted to hospital. NICE clinical guideline 92. London: National Institute for Health and Clinical Excellence.

http://www.nice.org.uk/guidance/CG92

This document has been authorised by the Department of Health
Gateway reference no: 10278

Department of Health

Department of Health

RISK ASSESSMENT FOR VENOUS THROMBOEMBOLISM (VTE)

**Mobility – all patients (tick one box)**	**Tick**		**Tick**		**Tick**
Surgical patient		Medical patient expected to have ongoing reduced mobility relative to normal state		Medical patient NOT expected to have significantly reduced mobility relative to normal state	
**Assess for thrombosis and bleeding risk below**				**Risk assessment now complete**	

**Thrombosis risk**			
**Patient related**	**Tick**	**Admission related**	**Tick**
Active cancer or cancer treatment		Significantly reduced mobility for 3 days or more	
Age > 60		Hip or knee replacement	
Dehydration		Hip fracture	
Known thrombophilias		Total anaesthetic + surgical time > 90 minutes	
Obesity (BMI >30 kg/m^2)		Surgery involving pelvis or lower limb with a total anaesthetic + surgical time > 60 minutes	
One or more significant medical comorbidities (eg heart disease;metabolic,endocrine or respiratory pathologies;acute infectious diseases; inflammatory conditions)		Acute surgical admission with inflammatory or intra-abdominal condition	
Personal history or first-degree relative with a history of VTE		Critical care admission	
Use of hormone replacement therapy		Surgery with significant reduction in mobility	
Use of oestrogen-containing contraceptive therapy			
Varicose veins with phlebitis			
Pregnancy or < 6 weeks post partum (see NICE guidance for specific risk factors)			

**Bleeding risk**			
**Patient related**	**Tick**	**Admission related**	**Tick**
Active bleeding		Neurosurgery, spinal surgery or eye surgery	
Acquired bleeding disorders (such as acute liver failure)		Other procedure with high bleeding risk	
Concurrent use of anticoagulants known to increase the risk of bleeding (such as warfarin with INR >2)		Lumbar puncture/epidural/spinal anaesthesia expected within the next 12 hours	
Acute stroke		Lumbar puncture/epidural/spinal anaesthesia within the previous 4 hours	
Thrombocytopaenia (platelets< 75x10^9/l)			
Uncontrolled systolic hypertension (230/120 mmHg or higher)			
Untreated inherited bleeding disorders (such as haemophilia and von Willebrand's disease)			

© Crown copyright 2010
301292 1p March 10
